# Clinical aspects of visceral leishmaniasis caused by *L. infantum* in adults. Ten years of experience of the largest outbreak in Europe: what have we learned?

**DOI:** 10.1186/s13071-019-3628-z

**Published:** 2019-07-24

**Authors:** Luis Horrillo, Alicia Castro, Belén Matía, Laura Molina, Jesús García-Martínez, Jerónimo Jaqueti, Isabel García-Arata, Eugenia Carrillo, Javier Moreno, José Manuel Ruiz-Giardin, Juan San Martín

**Affiliations:** 1 Área de Infecciosas, Servicio de Medicina Interna, Hospital Universitario de Fuenlabrada, Camino del Molino 2, 28942 Fuenlabrada, Madrid Spain; 2 Universidad Rey Juan Carlos, Avda. Atenas s/n, 28922 Alcorcón, Madrid Spain; 3 Área de Microbiología, Servicio de Laboratorio Clínico, Hospital Universitario de Fuenlabrada, Camino del Molino 2, 28942 Fuenlabrada, Madrid Spain; 4 Centro Nacional de Microbiología, WHO Collaborating Centre for Leishmaniasis, Majadahonda, Madrid Spain

**Keywords:** Visceral leishmaniasis, *Leishmania infantum*, Outbreak, Diagnosis, Therapy, Immunocompromised host, HIV

## Abstract

**Background:**

An outbreak of leishmaniasis caused by *Leishmania infantum* was declared in the southwest of the Madrid region (Spain) in June 2009. This provided a unique opportunity to compare the management of visceral leishmaniasis (VL) in immunocompetent adults (IC-VL), patients with HIV (HIV-VL) and patients receiving immunosuppressants (IS-VL).

**Methods:**

A cohort of adults with VL, all admitted to the Hospital Universitario de Fuenlabrada between June 2009 and June 2018, were monitored in this observational study, recording their personal, epidemiological, analytical, diagnostic, treatment and outcome variables.

**Results:**

The study population was made up of 111 patients with VL (10% HIV-VL, 14% IS-VL, 76% IC-VL). Seventy-one percent of the patients were male; the mean age was 45 years (55 years for the IS-VL patients, *P* = 0.017). Fifty-four percent of the IC-VL patients were of sub-Saharan origin (*P* = 0.001). Fever was experienced by 98% of the IC-VL patients *vs* 73% of the LV-HIV patients (*P* = 0.003). Plasma ferritin was > 1000 ng/ml in 77% of the IC-VL patients *vs* 17% of the LV-HIV patients (*P* = 0.007). Forty-two percent of patients fulfilled the criteria for haemophagocytic lymphohistiocytosis. RDT (rK39-ICT) serological analysis returned sensitivity and specificity values of 45% and 99%, respectively, and ELISA/iIFAT returned 96% and 89%, respectively, with no differences in this respect between patient groups. Fourteen (13.0%) patients with VL experienced treatment failure, eight of whom were in the IC-VL group. Treatment with < 21 mg/kg (total) liposomal amphotericin B (LAB) was associated with treatment failure in the IC-VL patients [*P* = 0.002 (OR: 14.7; 95% CI: 2.6–83.3)].

**Conclusions:**

IS-VL was more common than HIV-VL; the lack of experience in dealing with IS-VL is a challenge that needs to be met. The clinical features of the patients in all groups were similar, although the HIV-VL patients experienced less fever and had lower plasma ferritin concentrations. RDT (rK39-ICT) analysis returned a good specificity value but a much poorer sensitivity value than reported in other scenarios. The patients with HIV-VL, IS-VL and IC-VL returned similar serological results. Current guidelines for treatment seem appropriate, but the doses of LAB required to treat patients with HIV-VL and IS-VL are poorly defined.

## Background

Visceral leishmaniasis (VL) is the most severe clinical manifestation of disease caused by *Leishmania* parasites. In June 2009, an outbreak of leishmaniasis caused by *Leishmania infantum* was declared in the southwest of the Madrid region (Spain); the focus was an urban park in the town of Fuenlabrada. The incidence before the outbreak was around 0.2 cases/100,000 inhabitants in the above region, rising to 43.5/100,000 in Fuenlabrada during the outbreak [1]. With more than 700 cases declared by December 2016 (the date of the last official report [1]) the Fuenlabrada outbreak is the largest in Europe to date, and has not yet been declared over. Although most people affected during this outbreak have presented with cutaneous leishmaniasis, many patients have presented with VL and most of VL patients were immunocompetent and of all ages [2].

Large outbreaks involving zoonotic parasites such as *L. infantum* are very uncommon, especially in a European city [3]. The Fuenlabrada outbreak has some peculiarities. For the first time, hares appear to be the main reservoir (dogs, the usual reservoir for *L. infantum*, appear to have no role) [4]. Furthermore, it involves the ITS-LOMBARDI strain instead of the more common MON-1 strain; this new, poorly characterized strain was isolated from persons with different clinical manifestations of the disease during the outbreak [5]. The clinical behaviour of the disease in such an unusual scenario was therefore unknown. Indeed, we reported the appearance of cases of *L. infantum*-induced localized leishmanial lymphadenopathy (LLL) during the outbreak. Completely different to VL, this rare and fortunately benign clinical form is not described in clinical guidelines [6].

It was in 2010 that the WHO first recommended the management of VL to be individualised according to the causal species, the region of the world and patient immunological status {i.e. immunocompetent (IC-VL), co-infected with HIV (HIV-VL) or immunosuppressed (IS-VL) [7]}. These recommendations have been maintained in subsequent guidelines, and there have been no essential changes to recommendations on the management of VL in the Mediterranean area. However, recommendation levels regarding some aspects of diagnosis and treatment are low [3, 8, 9]. For instance, there is no certainty regarding the usefulness of the rapid diagnostic test (RDT) based on rK39 since the results reported have been highly variable by region [10]. In addition, the validity of serological tests for HIV-VL patients in the HAART era is unknown [11] and treatment recommendations for *L. infantum*-induced IC-VL, HIV-VL and IS-VL are based on small series of patients and even individual cases [3, 8, 9]. In this scenario of uncertainty, the aim of the present work was to describe our clinical experience in the management of such patients, with special attention paid to their immunological status. To our knowledge, this is the first time that personal, epidemiological, analytical, diagnostic, treatment and outcome variables for patients with IC-VL, HIV-VL and IS-VL have been compared for the same outbreak.

## Methods

### Design

This work was designed as a longitudinal observational study of a cohort of consecutive adult patients with VL treated at the Hospital Universitario de Fuenlabrada (HUF) from June 2009 to June 2018. A descriptive analysis was made of their clinical characteristics, the diagnostic methods employed, their treatment and the progress of their disease.

### Fuenlabrada hospital and the surrounding population

The HUF is the only public reference hospital for the city of Fuenlabrada. Located in the southwest of the Madrid region (Spain), its 400 beds serve a population of 221,986 people.

### Inclusion and exclusion criteria

All patients had to be at least 14 years of age at the time of diagnosis and fulfil the case definition for VL (see below). Patients who had received a solid organ transplant were excluded.

### Definitions

The case definition of VL was clinical manifestations compatible with the condition plus at least one of the following [7]: (i) positive parasitological test (optical microscopy of bone marrow aspirate, or blood/bone marrow PCR); and (ii) positive serological [RDT rK39-ICT and ELISA/iIFAT (enzyme-linked immunosorbent assay/indirect immunofluorescent antibody test)] test plus clinical response to treatment.

Patients with IC-VL were defined as those with VL with no apparent immunodeficiency, HIV-VL patients were defined as patients with VL plus chronic HIV infection, and IS-VL patients as those with VL receiving treatment with corticosteroids, methotrexate or anti-TNF drugs, regardless of the underlying disease.

Delay in diagnosis was described as the days elapsed between the patient reporting the onset of symptoms and a diagnosis being made. Treatment outcomes were described as [3]: (i) initial response: clinical improvement at the end of treatment; (ii) relapse: recurrence (meeting VL criteria once again) after initial response; (iii) definitive response/cured patient: absence of clinical symptoms 1 year after finishing treatment or re-treatment after relapse; and (iv) failure: lack of initial response and/or relapse.

Haemophagocytic lymphohistiocytosis (HLH) was deemed present when at least five of the eight diagnostic criteria re-defined in 2004 by the HLH Study Group were met [12]. Only six of the eight criteria were evaluated (the absence of NK activity and soluble CD25 could not be tested): (i) fever; (ii) splenomegaly; (iii) cytopenia (affecting ≥ 2 of 3 lineages in the peripheral blood); (iv) hypertriglyceridaemia and/or hypofibrinogenaemia; (v) hemophagocytosis in the bone marrow, spleen or lymph nodes; and (vi) ferritin ≥ 500 µg/l.

### Variables recorded and diagnostic tests performed

#### Epidemiological variables

The epidemiological variables recorded were age, gender, country of origin, ethnicity, delay in diagnosis, comorbidities (diabetes, cirrhosis, neoplasms), HIV and treatment with methotrexate, steroids or anti-TNF.

#### Clinical variables

The clinical variables recorded were symptoms at diagnosis: splenomegaly (examined physically or by ultrasonography/CT; defined as a spleen > 13 cm in cephalocaudal diameter), anaemia (haemoglobin < 12 mg/dl), leucopenia (< 4000 leucocytes/mm^3^), thrombocytopenia (< 150,000 platelets/mm^3^), C-reactive protein (CRP), erythrocyte sedimentation rate (ESR) and plasma ferritin.

#### Diagnostic variables and tests

Parasites were visualised by optical microscopy (bone marrow aspirate), cultivation (bone marrow aspirate) in Novy-MacNeal-Nicolle medium, or PCR-detected (*Leishmania* spp. DNA) in blood and bone marrow aspirate. All samples were sent to the National Center of Microbiology, ISCIII (Majadahonda, Madrid), for analysis.

Serological examination included immunochromatographic RDT based on antigen rK39-ICT, iIFAT and/or ELISA. The RDT (rK39-ICT) test was performed using colorimetric dipsticks, employing the SD *Leishmania* Ab kit (Standard Diagnostics, INC., Yongin-si, Gyeonggi-do, Korea) according to the manufacturer’s instructions. These dipsticks detect antibodies against the rK39 antigen. The results are available in less than 1 h. Our hospital laboratory began using this test in April 2011. ELISA was used to detect total antibodies against *Leishmania* using the *Leishmania* ELISA IgG + IgM kit (Vircell Microbiologists, Granada, Spain). This technique is considered as reliable as iIFAT and has been available at our hospital since April 2011. Before April 2011, samples were sent to the Microbiology Department of the Hospital Universitario Severo Ochoa for iIFAT testing using the *Leishmania* Indirect Immunofluorescence Antibody Test kit (Vircell Microbiologists, Granada, Spain). Titres ≥ 1:80 were considered positive.

#### Treatment and outcome variables

The following treatment and outcome variables were recorded: type of treatment, dose, adverse effects (creatinine > 0.5 mg/dl over baseline, chills), initial response, relapse, definitive response/cure, failure and death.

### Statistical analysis

Qualitative variables were characterized by their absolute and relative frequencies. Quantitative variables were analysed using the Kolmogorov–Smirnov test to determine if they fitted a normal distribution. Results are presented as the mean ± standard deviation (SD) for normally distributed variables, and as the medians plus interquartile range (IQR) for those that were not.

Results for categorical variables for the different patient groups were examined using the Chi-square test; when any frequency was < 5, Fisher’s exact test was used. Non-categorical variables were compared using either Student’s t-test (for parametric variables) or the Kruskal–Wallis test (for non-parametric variables).

Multivariate logistic regression was performed to determine the association of different variables with “failure” and “relapse”. The regression model included age, sex, sub-Saharan origin, delay in diagnosis, plasma ferritin, HLH, adequacy of liposomal amphotericin B (LAB) dose, compliance with secondary prophylaxis (patients with HIV-VL) and immunological status. Significance was set at *P* < 0.05. All calculations were performed using SPSS v.15.0 software for Windows.

## Results

### Distribution of cases by immunological status and year

Since the beginning of the outbreak, 111 patients have met the case definition criteria for VL. Fourteen patients clinically suspected of having VL returned positive serological results (1 by RDT rK39-ICT and 13 by ELISA) but did not meet the case definition criteria. Of these 14 patients, four had autoimmune disease, two were on immunosuppressant therapy, and for two a diagnosis of Q fever was made.

**Fig. 1:**
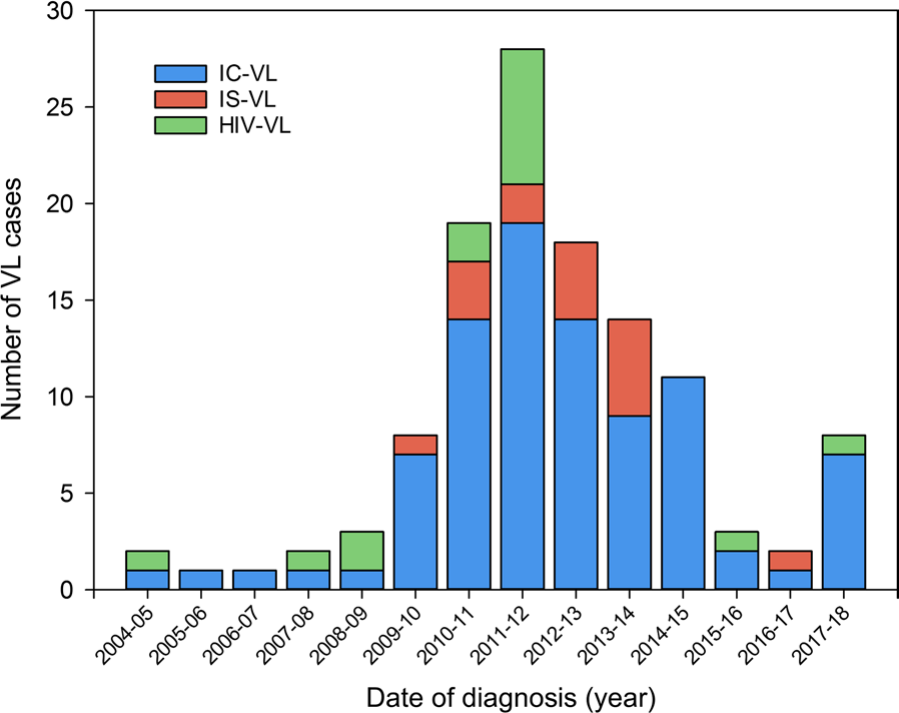
Distribution of VL patients diagnosed at the Hospital Universitario de Fuenlabrada since its opening. *Abbreviations*: IC-VL, visceral leishmaniasis in immunocompetent patients; HIV-VL, visceral leishmaniasis in patients with HIV; IS-VL, visceral leishmaniasis in immunosuppressed patients (receiving steroids, methotrexate, anti-TNF)

The 111 confirmed cases of VL were distributed as follows: 11 (10%) HIV-VL, 16 (14%) IS-VL and 84 (76%) IC-VL; Fig. 1 shows their diagnostic distribution by year compared to previous VL cases. Five (3.5%) IC-VL patients had liver cirrhosis and three (2.1%) had some type of active non-haematological malignancy.

Tables 1 and 2 show patient baseline characteristics at the time of HIV-VL or IS-VL diagnosis. No cases of liver tumoral disease or congenital immunodeficiency were detected (Table 2).

**Table 1:** Baseline characteristics of the patients with HIV (HIV-VL) at the time of VL diagnosis

Sex, age (years)	HIV risk factor^a^	Origin	Time between HIV and VL diagnoses	HCV co-infection	CD4	Viral load^b^	HAART
Male, 48	PDU	Spain	27 years	HCV	99	348,063	No
Male, 46	PDU	Spain	20 years	HCV	4	563,499	No
Male, 34	Hetero	Peru	3 years	No	29	288,439	No
Female, 45	Hetero	Eq. Guinea	4 years	No	305	< 20	TDF/FTC/EFV
Male, 48	PDU	Spain	16 years	HCV	46	660,099	No
Male, 37	Hetero	Nigeria	3 months	No	322	21,511	No
Female, 21	Vertical	Eq. Guinea	21 years	No	16	60,600	No
Male, 32	Hetero	Romania	20 days	No	59	143,400	No
Male, 36	Hetero	Nigeria	6 days	No	4	1,169,645	No
Male, 39	MSM	Poland	5 months	No	48	149,661	No
Male, 33	MSM	Moldova	2 days	No	40	1,610,000	No

^a^How the patients became infected by HIV

^b^Number of RNA viral copies/ml at the moment of VL diagnosis

*Abbreviations*: PDU, parenteral drug user; MSM, men who have sex with men; Hetero, heterosexual; CD4, number of CD4 cells/mm^3^ at the moment of VL diagnosis; HAART, highly active antiretroviral therapy; HCV, hepatitis C virus; TDF/FTC/EFV, tenofovir/emtricitabine/efavirenz

**Table 2:** Characteristics of the immunosuppressed patients (IS-VL) at the time of VL diagnosis

Sex, age (years)	Underlying disease	Origin	Time between starting immunosuppression and VL diagnosis	Corticosteroids (CE)^a^	Methotrexate	Anti-TNF	Others
Female, 68	RA	Spain	> 1 year	CE, low	Methotrexate	No	No
Male, 50	IBD	Spain	4 months	CE, intermediate	No	No	No
Male, 88	COPD	Spain	> 1 year	CE, high	No	No	No
Male, 64	Psoriatic arthritis	Spain	> 1 year	No	Methotrexate	No	No
Male, 40	UIP	Spain	> 1 year	CE, intermediate	No	No	Azathioprine
Female, 54	RA	Spain	> 1 year	CE, intermediate	Methotrexate	Etanercept	No
Female, 69	RA	Cuba	4 months	CE, intermediate	Methotrexate	No	No
Male, 54	Psoriatic arthritis	Spain	Mtx > 1 year, Eta 10 months	CE, low	Methotrexate	Etanercept	No
Male, 65	COPD	Spain	> 1 year	CE, high	No	No	No
Female, 44	IBD	Spain	> 1 year	No	No	Infliximab	Azathioprine
Female, 60	RA	Spain	> 1 year	No	Methotrexate	No	IL-20
Female, 47	IBD	Spain	16 months	No	No	Adalimumab	No
Female, 44	Psoriasis	Spain	> 1 year	No	Methotrexate	No	No
Male, 72	Giant-cell arteritis	Spain	> 1 year	CE, high	No	No	No
Male, 33	Tubulo-interstitial nephritis	Nigeria	5 months	CE, high	No	No	No
Male, 30	IgA nephropathy	Spain	> 1 year	CE, low	No	No	No

^a^ CE doses (prednisone equivalent): low dose, below 5 mg/day; intermediate dose, 5–10 mg/day; high dose, above 30 mg/day

*Abbreviations*: RA, rheumatoid arthritis; IBD, inflammatory bowel disease; COPD, chronic obstructive pulmonary disorder; UIP, usual interstitial pneumonia; CE, corticosteroids; IL-20, monoclonal antibody anti-IL-20

### Clinical features

**Fig. 2:**
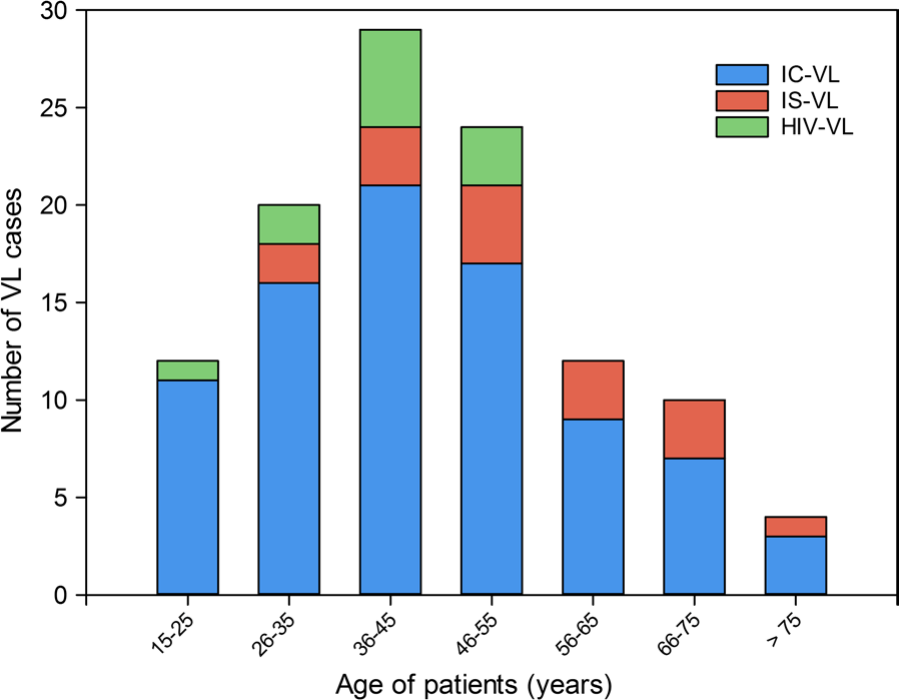
Age distribution of VL patients during the outbreak. *Abbreviations*: IC-VL, visceral leishmaniasis in immunocompetent patients; HIV-VL, visceral leishmaniasis in patients with HIV; IS-VL, visceral leishmaniasis in immunosuppressed patients (receiving steroids, methotrexate, anti-TNF)

Table 3 and Fig. 2 show the clinical characteristics of the patients according to their immunological status.

**Table 3:** Clinical characteristics according to immunological status

	IC-VL (*n* = 84)	HIV-VL (*n* = 11)	IS-VL (*n* = 16)	Total VL (*n* = 111)	*P-v*alue
Male (%)	73	82	56	71	0.297
Age (mean ± SD, years)	44.0 ± 16.5	38.2 ± 8.0	54.8 ± 16.0	45.0 ± 16.9	ANOVA: *F*_(2, 110)_ = 6.22, *P* = 0.017*
Immigrants (%)	58	73	13	53	*χ*^2^ = 13.22, *df* = 2, *P* = 0.001*
Sub-Saharan origin (%)	54	36	13	46	*χ*^2^ = 9.58, *df* = 2, *P* = 0.008*
Diabetes (%)	16	9	13	14	0.828
Distance to park (mean ± SD, m)	922 ± 566	928 ± 701	917 ± 544	921 ± 569	0.999
Delay of diagnosis^a^ (median (IQR), days)	18.5 (14–30)	21 (16–60)	24 (10–60)	20 (13–30)	0.640
Fever (%)	98	73	94	95	*χ*^2^ = 11.81, *df* = 2, *P* = 0.003*
Asthenia (%)	49	46	69	51	0.315
Weight loss (%)	40	36	31	39	0.786
Anorexia (%)	33	36	25	32	0.784
Cough (%)	31	36	31	32	0.836
Cephalea (%)	35	9	19	30	0.113
Vomiting (%)	21	9	0	16	0.056
Abdominal pain (%)	15	18	12	15	0.762
Odynophagia (%)	10	9	6	9	0.906
Diarrhoea (%)	6	18	13	8	0.314
Dyspnoea (%)	7	9	0	6	0.512
Weight (mean ± SD, kg)	78.5 ± 19.1	68.3 ± 18.0	76.4 ± 20.1	77.1 ± 19.2	0.254
Clinical splenomegaly (%)	29	46	31	31	0.537
Radiological splenomegaly, *n*/T (%)	75/79 (95)	10/11 (91)	13/15 (87)	93	0.472
Spleen size (mean ± SD, cm)	15.7 ± 2.0	15.9 ± 2.1	15.2 ± 2.32	15.7 ± 2.0	0.627
Anaemia (haemoglobin < 12 mg/dl) (%)	87	82	94	87	0.599
Haemoglobin (mean ± SD, mg/dl)	10.2 ± 1.7	10.9 ± 1.3	9.4 ± 1.5	10.2 ± 1.7	ANOVA: *F*_(2, 106)_ = 3.24, *P* = 0.043*
Leukopenia (< 4000/mm^3^) (%)	90	82	94	90	0.587
Leucocytes (mean ± SD, mm^3^)	2715 ± 902	2513 ± 1208	2470 ± 1083	2658 ± 958	0.565
Thrombocytopenia (< 150,000/mm^3^) (%)	93	91	100	94	0.512
Platelets (mean ± SD, mm^3^)	93,379 ± 41,771	103,364 ± 38,263	77,875 ± 29,132	92,111 ± 40,077	0.228
CRP (mean ± SD, mg/dl)	12.2 ± 7.3	8.3 ± 6.8	9.2± 6.9	11.3 ± 7.3	0.112
CRP > 10 mg/dl	56	36	38	51	0.225
Ferritin (median (IQR), ng/ml)	2264 (914–6368.5)	712 (469–1098.5)	1854 (1146–5369)	1969 (838–5784)	Kruskal-Wallis H-test: *χ*^2^ = 6.31, *df* = 2, *P* = 0.043*
Ferritin > 1000 ng/ml, *n*/T (%)	56/73 (77)	1/6 (17)	11/14 (79)	68/93 (73)	*χ*^2^ = 11.81, *df* = 2, *P* = 0.005*
ESR, *n*/T (mean ± SD, mm/h)	25/44 (73 ± 32)	3/6 (73 ± 26)	2/11 (60 ± 35)	30/61 (71 ±32)	0.494
ESR > 70 mm/h, *n*/T (%)	25/44 (57)	3/6 (50)	2/11 (18)	30/61 (49)	0.072
Triglycerides (mean ± SD, mg/dl)	220 ± 81	170 ± 66	203 ± 64	211 ± 78	0.124
HLH^b^, *n* (%)	31 (37)	2 (18)	9 (56)	42 (38)	0.129
Auto-antibody positive, *n*/T (%)	13/44 (30)	2/4 (50)	3/14 (21)	18/62 (29)	0.535
Serology infectious diseases^c^ positives, *n*/T (%)	25/55 (46)	1/6 (17)	5/10 (50)	31/71 (44)	0.366

^a^Time in days from when patient noticed symptoms to diagnosis

^b^HLH criteria are shown in Table 4

^c^IgG positive for other infectious diseases: *Borrelia burgdorferi* 11, *Coxiella burnetti* 10, *Parvovirus* 6, *Mycoplasma* 4, *Chlamydophila* 4, *Rickettsia* 4, Syphilis RPR 2

*Abbreviations*: n, sample size for each group; IC-VL, visceral leishmaniasis in immunocompetent patients; HIV-VL, visceral leishmaniasis in patients with HIV; IS-VL, visceral leishmaniasis in immunosuppressed patients (receiving steroids, methotrexate, anti-TNF), SD, standard deviation; IR, interquartile range; *n*/T, number of positives/total number tested; CRP, C-reactive protein; ESR, erythrocyte sedimentation rate; HLH: haemophagocytic lymphohistiocytosis

*P < 0.05

The majority of affected immigrants came from Equatorial Guinea (*n* = 24) and Nigeria (*n* = 14), all of whom had been living in Spain for over 6 months. No significant differences were recorded for this group with respect to the mean distance to the focus of the outbreak (mean ± SD 908 ± 590 *vs* 955 ± 551 m for the remaining patients; ANOVA: *F*_(1, 99)_ = 0.058, *P* = 0.810) or body weight before the start of treatment (79.5 ± 18.7 *vs* 75.4 ± 19.5 kg for the remaining patients; ANOVA: *F*_(1, 105)_ = 1.313, *P* = 0.26).

Overall, 38% of the patients (56% of the IS-VL patients, 18% of the HIV-VL and 37% of the IC-VL patients) fulfilled the criteria for HLH (Table 3). Table 4 shows the results for the HLH criteria assessed.

**Table 4:** HLH criteria assessed

	IC-VL (*n* = 84)	HIV-VL (*n* = 11)	IS-VL (*n* = 16)	Total (*n* = 111)	*P-*value
Fever (%)	98	73	94	95	*χ*^2^ = 11.81, *df* = 2, *P* = 0.003*
Radiological splenomegaly (%)	95	91	87	93	0.472
Cytopenia (at least 2 blood cell lines) (%)	94	91	94	94	0.930
Hypertriglyceridemia (> 265 mg/dl) (%)	23	9	25	22	0.552
Hemophagocytosis in bone marrow (%)	50	22	75	52	*χ*^2^ = 6.69, *df* = 2, *P* = 0.035*
Ferritin > 500 mg/l (%)	86	78	93	86	0.551

*Abbreviations*: n, sample size for each group; IC-VL, visceral leishmaniasis in immunocompetent patients; HIV-VL, visceral leishmaniasis in patients with HIV; IS-VL, visceral leishmaniasis in immunosuppressed patients (receiving steroids, methotrexate, anti-TNF)

*P < 0.05

### Diagnosis

Table 5 shows the results of the diagnostic tests. Before the introduction of the RDT (rK39-ICT) test, the median delay in diagnosis was 30 days (IQR: 18.5–82.5). After the introduction of the test it decreased to a median of 16 days (IQR: 10–28) (Kruskal–Wallis H-test: *χ*^2^ = 5.094, *df *= 1, *P* = 0.024).

**Table 5:** Diagnostic test results

	IC-VL (*n* = 84)	HIV-VL (*n* = 11)	IS-VL (*n* = 16)	Total (*n* = 111)	*P-*value
RDT (rk39-ICT) (*n*/T, %)	26/62 (42)	5/10 (50)	7/12 (58)	38/84 (45)	0.550
ELISA/iIFAT (*n*/T, %)	77/81 (95)	11/11 (100)	16/16 (100)	104/108 (96)	0.500
Optical microscopy (bone marrow) (*n*/T, %)	32/78 (41)	5/10 (50)	8/16 (50)	45/104 (43)	0.726
Culture (bone marrow) (*n*/T, %)	6/28 (21)	3/3 (100)	3/6 (43)	12/37 (32)	*χ*^2^ = 8.64, *df* = 2, *p* =0.013*
PCR (bone marrow) (*n*/T, %)	67/73 (92)	9/10 (90)	16/16 (100)	92/99 (93)	0.474
PCR (blood) (*n*/T, %)	21/25 (84)	4/4 (100)	7/8 (88)	32/37 (87)	0.682

*Abbreviations*: RDT (rK39-ICT), rapid diagnostic test, immunochromatographic test based on rK39 antigen; n, sample size for each group; IC-VL, visceral leishmaniasis in immunocompetent patients; HIV-VL, visceral leishmaniasis in patients with HIV; IS-VL, visceral leishmaniasis in immunosuppressed patients (receiving steroids, methotrexate, anti-TNF); *n*/T, number of positives/total number of tests performed

### Treatment and outcome

Three patients with IC-VL were not treated, two with IC-VL were lost to follow-up, and one died due to complications of advanced cirrhosis before starting treatment. A total of 108 patients therefore received treatment, 104 with liposomal amphotericin B (LAB) and four with amphotericin B lipid complex (LABC) (doses shown in Table 6). All HIV-VL patients received HAART and secondary prophylaxis for VL after their VL diagnosis.

**Table 6:** Dose and clinical outcome in patients with VL according to their immunological status

LAB dose^a^ (mg/kg)	VL treated (*n* = 108)	Relapses (*n* = 14, 13.0%)
IC-VL^b^	81	8 (9.9%)
15	2	1 (50.0%)
18	5	3 (60.0%)
21	63 LAB + 2 ABCL	3 (4.6%)
30	5 LAB + 1 ABCL	1 (16.7%)^c^
HIV-VL	11	3 (27.3%)
21	1	1 (100%)^d^
30	4	2 (50.0%)^e^
40	6	0
IS-VL	16	3 (18.8%)
21	3 LAB + 1 ABCL	1 (25.0%)^f^
30	4	1 (25.0%)^c^
40	8	1 (12.5%)

^a^Usual LAB standard dose (see text): IC-VL 18–21 mg/kg; HIV-VL 30–40 mg/kg; IS-VL 21–40 mg/kg

^b^3 IC-VL patients with solid neoplasms were treated with 21, 30 and 40 mg/dl each, with no relapses

^c^Relapsed and died during retreatment

^d^This patient was clinically cured after treatment but voluntarily stopped secondary prophylaxis and relapsed

^e^One patient was clinically cured after treatment but voluntarily stopped secondary prophylaxis and relapsed

^f^Lack of initial response, cured after retreatment

*Abbreviations*: LAB, liposomal B amphotericin; ABLC, amphotericin B lipid complex; IC-VL, visceral leishmaniasis in immunocompetent patients; HIV-VL, visceral leishmaniasis in patients with HIV; IS-VL, visceral leishmaniasis in immunosuppressed patients (receiving steroids, methotrexate, anti-TNF)

An increase in creatinine of ≥ 0.5 mg/dl over baseline was observed in 35 patients (32%), but was reversible in all cases. Chills were recorded in 10 patients (9%). No significant differences in adverse effects were seen between the patient groups.

All but one patient met the initial response criteria (99%). The median follow-up of all treated patients was 316 weeks (IQR: 216–370), with no significant differences with respect to immunological status. Fourteen treatment failures (12.9%) were noted, 13 relapses (12%) and one lack of initial response (0.9%) (Table 6). Only four of the 14 patients who failed met the criteria for HLH (29%). No association was seen between meeting the criteria for HLH and relapse (*P* = 0.315). Table 7 shows the detailed characteristics of the eight IC-VL patients who relapsed.

**Table 7:** Clinical features of IC-VL patients who relapsed

Sex, age (years)	Origin	Sub-Saharan^a^	Days to relapse	Drug	Dose (mg/kg)	Retreatment dose (mg/kg)	Status 6 months after retreatment
Male, 38	Spain	No	270	LAB	15	35	Cured
Male, 95	Spain	No	30	LAB	18	30	Cured
Male, 15	Spain	Yes	60	LAB	18	30	Cured
Female, 37	Eq. Guinea	Yes	115	LAB	18	21	Cured
Female, 64	Eq. Guinea	Yes	97	ABLC	21	30 (LAB)	Cured
Male, 34	Eq. Guinea	Yes	165	LAB	21	20 (LAB + miltefosine)	Cured
Male, 39	Nigeria	Yes	71	LAB	21	40	Cured
Male, 51	Spain	No	190	LAB	30	12 (exitus cirrhosis)	Exitus

^a^Relapses in persons of sub-Saharan origin with IC-VL: 11.9% (5/42) *vs* 7.9% (3/38), *P* = 0.414

*Abbreviations*: LAB, liposomal B amphotericin; ABLC, amphotericin B lipid complex

Multivariate analysis identified treatment failure and relapse to be associated with doses of < 21 mg/kg in IC-VL patients (OR: 14.7; 95% CI: 2.6–83.3; *P* = 0.002) and non-compliance with prophylaxis in HIV-VL (OR: 7.2; 95% CI: 1.5–34.2; *P* = 0.013).

## Discussion

### Clinical features

The epidemiological characteristics of patients with VL in Fuenlabrada have undergone no substantial changes since the outbreak was declared ten years ago [2]. The present sample is dominated by males, as is the case for most VL case series [13–16], and contains patients of all ages (up to 95 years), as might be expected from data for epidemic outbreaks in non-endemic areas. In previous IC-VL series, male children dominated the sample, both in Spain [17] and in Italy [13, 18–20]. These Italian series from more than 20 years ago still form the basis of current treatment guidelines for VL, including for adults, in the Mediterranean area. In the present outbreak, the patients with IS-VL were older. A disproportionately high number of sub-Saharan immigrants with VL has been recorded since the beginning of the outbreak, despite the fact that the foreign population makes up only 13% of Fuenlabrada’s population [2], perhaps due to genetic factors [6]. The present results suggest that they were not infected in their countries of origin, and no differences were seen between them and the remaining patients in terms of the distance they lived from the focus of the outbreak.

Fever, splenomegaly and pancytopenia are the main clinical criteria of VL [3, 7], and affected > 90% of the patients in the present study. For many, cough, headache (in 30%), vomiting and abdominal pain were recorded, non-specific symptoms that hinder making a differential diagnosis. Certainly, thrombocytopenia, splenomegaly and non-specific focalizing symptoms can be confused with viral syndromes, or malaria in the case of sub-Saharan patients, or immunosuppressant-induced pancytopenia in the case of IS-VL patients. In areas where VL is little expected, diagnosis can become difficult, as revealed by the high median diagnostic delay in the first years of the outbreak.

Fever and plasma ferritin were the only clinical features that were less frequent in patients with HIV-VL. A lower proportion of patients with fever has been described in transplanted patients with VL [21]. This has been related to the incapacity of the humoral immune system to act against the parasite. However, the clinical presentation of disease was similar across the three patient groups. Some atypical forms, such as mucosal leishmaniasis and asymptomatic carriers, can be more common in patients co-infected with HIV, but in our experience the clinical presentation of HIV-VL was similar, in most cases, to that of IC-VL [11].

A large number of patients met the criteria for HLH, a syndrome associated with very high mortality [12]. However, no higher mortality nor risk of relapse was seen in the present work; indeed, the response to treatment for VL was good. In the Fuenlabrada setting, VL needs to be ruled out in all cases of HLH given its prognostic implications [22]. In the IC-VL and IS-VL patients, plasma ferritin was notably elevated; indeed, such concentrations are associated with very few diseases and in the present context can be taken as a relatively specific sign of VL.

A high percentage (30%) of patients were positive for auto-antibodies (commonly found in patients with VL) and for antibodies to other infectious agents (especially *Borrelia* and *Coxiella*) (45%). VL can therefore be initially confused with autoimmune disease [23], and certainly the cross-reactivity of these auto-antibodies with the histone protein of *Leishmania* [24] has been described. Thus, patients with autoimmune disease but without VL may test positive for *Leishmania* serology in ELISA. Indeed, four of the present patients had false positive serology results but did not meet the case definition criteria.

### Diagnosis

RDT based on rK39 was introduced during the outbreak as a first step test in the diagnosis of VL. Combining the RDT test with conventional testing is now recommended in the WHO European guidelines 2017 [3], but this was not the case in 2010 [7]. Although RDT has been reported as a sensitive and specific test (> 90% for both), the results returned have been highly variable by region [10]. The two studies performed in Europe reported sensitivities of between 52 and 100% [25, 26]. Its reliability for detecting *L. infantum* in Europe is therefore not clear. RDT (rK39-ICT) returned positive results for only 50% of patients with actual VL, although it had a positive predictive value (PPV) of 99%. Thus, a negative test cannot rule out VL, but a positive test for a patient in whom VL is clinically suspected is almost confirmatory. Conventional iIFAT and ELISA returned positive results for 96% of patients with VL, but at least 13 patients had false positive results (PPV 89%). This loss of specificity is not surprising in the context of an epidemic in which there may be many asymptomatic patients exposed to *Leishmania* who return a positive serological test. Thus, according to the present results, a patient with suspected VL who returned both positive RDT (rK39-ICT) and ELISA/iIFAT tests (regardless of the antibody titre) could be treated without the need for any additional test, while another disease should be sought in a patient returning two negative results. The WHO guidelines recommend that, where there is disagreement, a parasitological test is advisable, but in our experience this disagreement always involves a negative RDT (rK39-ICT) and a positive ELISA/iIFAT test.

No serological differences were detected between the IC-VL, IS-VL and HIV-VL patients. Based on older series, serological results are usually understood to be the least reliable for diagnostic purposes in patients with HIV-VL [11]. However, a recent meta-analysis detected better results for the most current series [27]. This might be explained in that these older series involved patients who were infected *via* the sharing of needles. Only three of the present HIV-VL patients acquired HIV *via* the parenteral route, and even in these patients, HIV was diagnosed at least 16 years before VL. It is therefore likely that all were infected *via* the normal vector. To examine this further, clinical and epidemiological data should be included in future studies on the serology of HIV-VL patients.

### Treatment and outcome

The treatment of choice for *L. infantum*-induced IC-VL is LAB (18–21 mg/kg total), but the recommendation level is low [3, 8]: no double-blind randomised studies comparing different treatment regimens have been undertaken, the case series on which this treatment choice is based are > 20 years old, and the data extracted from them refer largely to Italian children [13, 18, 19, 28]. In one of these studies, 15 mg/kg LAB cured 90% of the patients [13]. Given the renal toxicity of this drug, two of the present IC-VL patients were treated with 15 mg/kg. A cure rate of 100% was hoped for, but one patient relapsed. In addition, five IC-VL patients were treated with a dose of 18 mg/kg, as recommended by current guidelines [7], and three of these (60%) relapsed. Thus, an unexpectedly high relapse rate was observed with the < 21 mg/kg dose, which was in fact the only factor associated with relapse in the IC-VL patients. One might hypothesise about the virulence of the strain [29] or whether there are higher relapse rates among sub-Saharan patients, but the differences are not statistically significant. In our routine practice we strongly recommend the use of the standard regimen approved by the FDA for adults, i.e. 3 mg/kg/day on days 1–5, 14 and 21 in adults with IC-VL [8], avoiding other regimens and doses based on results obtained in children [13, 20].

Some guidelines recommended that patients with IC-VL should be followed up for 6–12 months [7, 8]. During the outbreak, two of our patients (25%) relapsed after six months; it would therefore seem sensible to monitor patients for up to 12 months [3].

Only three patients with active VL died during the outbreak: one IS-VL and two IC-VL patients. The latter two had severe chronic liver disease and were in a pre-transplant situation; the main cause of their deaths was deemed to be liver disease. However, the patients who also had cancer responded well, with no relapses.

Most guidelines recommend a higher dose of LAB (40 mg/kg) for patients co-infected with *Leishmania* and HIV, but the level of evidence for this is very low [3, 8]. A 30 mg/kg dose has also been recommended [7]. These recommendations were initially based on two case series, one of ten patients [30] and one of five patients [31], collated during the pre-HAART era. In both studies the initial response was good, but the percentage of relapses was high since secondary prophylaxis was not provided [32]. Although later, non-randomized studies that included HAART and secondary prophylaxis with LAB endorsed this strategy [33], the dose has not been re-evaluated despite some case series studies reporting an initial response with 15 mg/kg [34], 22 mg/kg [35] and 30 mg/kg [18]. The present HIV-VL patients had a good initial response to both the 30 and 40 mg/kg doses, so these lower doses seem appropriate. With current HAART, patients may certainly require less than 30 mg/kg, and secondary prophylaxis could likely be safely avoided in selected patients [36]. Multicentre trials to investigate the treatment of HIV-VL have been called for in different reviews [9, 33].

Finally, the IS-VL patients formed a heterogeneous group. It has been reported that steroids, methotrexate and anti-TNF drugs may favour the appearance of VL (these patients are considered immunosuppressed) [9, 37, 38]. Current recommendations suggest they should be managed in a manner similar to IC-VL patients, but this is based on evidence provided by individual cases [3, 8, 9]. The present IS-VL patients responded similarly to doses of 21 and 40 mg/kg LAB, and no factor associated with the few relapses recorded could be identified. More information on the treatment of such patients is required.

The main limitation of this work is its observational nature. For any conclusions to be drawn regarding the diagnostic tests performed or different treatment regimens tried, comparative clinical trials would need to be performed. In addition, the data were collected during an epidemic outbreak, and the sample excludes children and the recipients of solid organ transplants (to whose populations the results cannot be extrapolated).

## Conclusions

IS-VL was more frequent than HIV-VL; the lack of experience in dealing with IS-VL is a challenge that needs to be met. Given the present setting, it was deemed always appropriate to consider VL in the differential diagnosis of fever, splenomegaly, pancytopenia and very high plasma ferritin. The clinical features of the patients in each group were similar, although HIV-VL patients had less fever and lower plasma ferritin. RDT (rK39-ICT) was found to be highly specific as a diagnostic test, but much less sensitive than reported in other scenarios; however, a combination of positive RDT and ELISA or iIFAT tests is enough to warrant the start of treatment. The serology of HIV-VL, IS-VL and IC-VL patients was similar. Current guidelines for treatment seem appropriate, but the doses of LAB requited to treat patients with HIV-VL and IS-VL are poorly defined; more information is needed. The standard regimen of LAB (3 mg/kg/day on days 1–5, 14 and 21) seems appropriate for the treatment of adults with IC-VL, but not lower doses. Developing methods to help predict relapse would be very useful.

## Data Availability

Data supporting the conclusions of this article are included within the article. The datasets used and/or analyzed during the present study are available from the corresponding author upon request.

## Abbreviations

VL: visceral leishmaniasis in immunocompetent adults
IC-VL: visceral leishmaniasis in immunocompetent adults
HIV-VL: visceral leishmaniasis in patients with HIV
IS-VL: visceral leishmaniasis in patients receiving immunosuppressants
RDT (rK39-ICT): rapid diagnostic test, immunochromatographic test based on rK39 antigen
LAB: liposomal amphotericin B
HAART: highly active antirretroviral therapy
HUF: Hospital Universitario de Fuenlabrada
HLH: haemophagocytic lymphohistiocytosis

## References

[1] Dirección General de Salud Pública. Leishmaniasis en la Comunidad de Madrid, 2015. http://www.madrid.org/bvirtual/BVCM017837.pdf. Accessed 16 Dec 2018.

[2] Arce A, Estirado A, Ordobas M, Sevilla S, García N, Moratilla L (2013). Re-emergence of leishmaniasis in Spain: community outbreak in Madrid, Spain, 2009 to 2012. Euro Surveill..

[3] WHO/Regional Office for Europe. Manual on case management and surveillance of the leishmaniases in the WHO European Region. 2017. http://www.who.int/leishmaniasis/resources/978-92-89052-51-1/en/. Accessed 22 Apr 2018.

[4] Molina R, Jiménez MI, Cruz I, Iriso A, Martín-Martín I, Sevillano O (2012). The hare (*Lepus granatensis*) as potential sylvatic reservoir of *Leishmania infantum* in Spain. Vet Parasitol..

[5] Chicharro C, Llanes-Acevedo IP, García E, Nieto J, Moreno J, Cruz I (2013). Molecular typing of *Leishmania infantum* isolates from a leishmaniasis outbreak in Madrid, Spain, 2009 to 2012. Euro Surveill..

[6] Horrillo L, San Martín JV, Molina L, Madroñal E, Matía B, Castro A (2015). Atypical presentation in adults in the largest community outbreak of leishmaniasis in Europe (Fuenlabrada, Spain). Clin Microbiol Infect..

[7] WHO Expert Committee on the Control of the Leishmaniases. Control of the leishmaniases: report of a meeting of the WHO Expert Committee on the Control of Leishmaniases, Geneva, 22–26 March 2010. https://apps.who.int/iris/bitstream/handle/10665/44412/WHO_TRS_949_eng.pdf;jsessionid=92DC989D367A1E7AD2FA0A7E8A56FB90?sequence=1. Accessed 22 Apr 2018.

[8] Aronson N, Herwaldt BL, Libman M, Pearson R, Lopez-Velez R, Weina P (2017). Diagnosis and treatment of leishmaniasis: clinical practice guidelines by the Infectious Diseases Society of America (IDSA) and the American Society of Tropical Medicine and Hygiene (ASTMH). Am J Trop Med Hyg..

[9] van Griensven J, Carrillo E, López-Vélez R, Lynen L, Moreno J (2014). Leishmaniasis in immunosuppressed individuals. Clin Microbiol Infect..

[10] Boelaert M, Verdonck K, Menten J, Sunyoto T, van Griensven J, Chappuis F (2014). Rapid tests for the diagnosis of visceral leishmaniasis in patients with suspected disease. Cochrane Database Syst Rev..

[11] Alvar J, Aparicio P, Aseffa A, Den Boer M, Cañavate C, Dedet J-P (2008). The relationship between leishmaniasis and AIDS: the second 10 years. Clin Microbiol Rev..

[12] Henter J-I, Horne A, Aricó M, Egeler RM, Filipovich AH, Imashuku S (2007). HLH-2004: diagnostic and therapeutic guidelines for hemophagocytic lymphohistiocytosis. Pediatr Blood Cancer..

[13] Davidson RN, Di Martino L, Gradoni L, Giacchino R, Gaeta GB, Pempinello R (1996). Short-course treatment of visceral leishmaniasis with liposomal amphotericin B (AmBisome). Clin Infect Dis..

[14] Leta S, Dao THT, Mesele F, Alemayehu G (2014). Visceral leishmaniasis in Ethiopia: an evolving disease. PLoS Negl Trop Dis..

[15] Reis LLD, Balieiro AAS, Fonseca FR, Gonçalves MJF (2017). Changes in the epidemiology of visceral leishmaniasis in Brazil from 2001 to 2014. Rev Soc Bras Med Trop..

[16] Sinha PK, van Griensven J, Pandey K, Kumar N, Verma N, Mahajan R (2011). Liposomal amphotericin B for visceral leishmaniasis in human immunodeficiency virus-coinfected patients: 2-year treatment outcomes in Bihar. India. Clin Infect Dis..

[17] Ramos JM, Clavijo A, Moral L, Gavilan C, Salvador T, González de Dios J (2018). Epidemiological and clinical features of visceral leishmaniasis in children in Alicante Province, Spain. Paediatr Int Child Health..

[18] Davidson RN, Di Martino L, Gradoni L, Giacchino R, Russo R, Gaeta GB (1994). Liposomal amphotericin B (AmBisome) in Mediterranean visceral leishmaniasis: a multi-centre trial. Q J Med..

[19] Syriopoulou V, Daikos GL, Theodoridou M, Pavlopoulou I, Manolaki AG, Sereti E (2003). Two doses of a lipid formulation of amphotericin B for the treatment of Mediterranean visceral leishmaniasis. Clin Infect Dis..

[20] Cascio A (2004). A 6 day course of liposomal amphotericin B in the treatment of infantile visceral leishmaniasis: the Italian experience. J Antimicrob Chemother..

[21] Antinori S, Cascio A, Parravicini C, Bianchi R, Corbellino M (2008). Leishmaniasis among organ transplant recipients. Lancet Infect Dis..

[22] Hernández-Jiménez P, Díaz-Pedroche C, Laureiro J, Madrid O, Martín E, Lumbreras C (2016). Linfohistiocitosis hemofagocítica: análisis de 18 casos. Med Clin..

[23] Ortiz M, Mon C, Herrero JC, Oliet A, Rodríguez I, Ortega O (2015). Glomerulonephritis and cryoglobulinemia: first manifestation of visceral leishmaniasis. Clin Nephrol..

[24] Lakhal S, Benabid M, Sghaier IB, Bettaieb J, Bouratbine A, Galai Y (2015). The sera from adult patients with suggestive signs of autoimmune diseases present antinuclear autoantibodies that cross-react with *Leishmania infantum* conserved proteins: crude *Leishmania* histone and soluble *Leishmania* antigens [corrected]. Immunol Res.

[25] Brandonisio O, Fumarola L, Maggi P, Cavaliere R, Spinelli R, Pastore G (2002). Evaluation of a rapid immunochromatographic test for serodiagnosis of visceral leishmaniasis. Eur J Clin Microbiol Infect Dis..

[26] Varani S, Ortalli M, Attard L, Vanino E, Gaibani P, Vocale C (2017). Serological and molecular tools to diagnose visceral leishmaniasis: 2-years’ experience of a single center in Northern Italy. PLoS ONE..

[27] Cota GF, de Sousa MR, Demarqui FN, Rabello A (2012). The diagnostic accuracy of serologic and molecular methods for detecting visceral leishmaniasis in HIV infected patients: meta-analysis. PLoS Negl Trop Dis..

[28] di Martino L, Davidson RN, Giacchino R, Scotti S, Raimondi F, Castagnola E (1997). Treatment of visceral leishmaniasis in children with liposomal amphotericin B. J Pediatr..

[29] Domínguez-Bernal G, Jiménez M, Molina R, Ordóñez-Gutiérrez L, Martínez-Rodrigo A, Mas A (2014). Characterisation of the *ex vivo* virulence of *Leishmania infantum* isolates from *Phlebotomus perniciosus* from an outbreak of human leishmaniosis in Madrid. Spain. Parasit Vectors..

[30] Russo R, Nigro LC, Minniti S, Montineri A, Gradoni L, Caldeira L (1996). Visceral leishmaniasis in HIV infected patients: treatment with high dose liposomal amphotericin B (AmBisome). J Infect..

[31] Laguna F, Torre-Cisneros J, Moreno V, Villanueva JL, Valencia E (1995). Efficacy of intermittent liposomal amphotericin B in the treatment of visceral leishmaniasis in patients infected with human immunodeficiency virus. Clin Infect Dis..

[32] Sundar S, Chakravarty J (2010). Liposomal amphotericin B and leishmaniasis: dose and response. J Glob Infect Dis..

[33] Molina I, Falcó V, Crespo M, Riera C, Ribera E, Curran A (2007). Efficacy of liposomal amphotericin B for secondary prophylaxis of visceral leishmaniasis in HIV-infected patients. J Antimicrob Chemother..

[34] Lebrato JC, García ET, Quero JH, Escobar T (2001). Tratamiento de la coinfección VIH-leishmaniasis visceral con un nuevo régimen de anfotericina B liposomal (AMB-L). Med Clin..

[35] Torre-Cisneros J, Villanueva JL, Kindelan JM, Jurado R, Sanchez-Guijo P (1993). Successful treatment of antimony-resistant visceral leishmaniasis with liposomal amphotericin B in patients infected with human immunodeficiency virus. Clin Infect Dis..

[36] Castro A, Carrillo E, San Martín JV, Botana L, Molina L, Matía B (2016). Lymphoproliferative response after stimulation with soluble *Leishmania* antigen (SLA) as a predictor of visceral leishmaniasis (VL) relapse in HIV+ patients. Acta Trop..

[37] Zanger P, Kötter I, Kremsner PG, Gabrysch S (2012). Tumor necrosis factor alpha antagonist drugs and leishmaniasis in Europe. Clin Microbiol Infect..

[38] Pagliano P, Ascione T, Di Flumeri G, Boccia G, De Caro F (2016). Visceral leishmaniasis in immunocompromised: diagnostic and therapeutic approach and evaluation of the recently released IDSA guidelines. Infez Med..

